# Silencing *SlMED18*, tomato Mediator subunit 18 gene, restricts internode elongation and leaf expansion

**DOI:** 10.1038/s41598-018-21679-1

**Published:** 2018-02-19

**Authors:** Yunshu Wang, Zongli Hu, Jianling Zhang, XiaoHui Yu, Jun-E. Guo, Honglian Liang, Changguang Liao, Guoping Chen

**Affiliations:** Laboratory of molecular biology of tomato, Bioengineering College, Chongqing University, Chongqing, People’s Republic of China

## Abstract

Mediator complex, a conserved multi-protein, is necessary for controlling RNA polymerase II (Pol II) transcription in eukaryotes. Given little is known about them in tomato, a tomato Mediator subunit 18 gene was isolated and named *SlMED18*. To further explore the function of *SlMED18*, the transgenic tomato plants targeting *SlMED18* by RNAi-mediated gene silencing were generated. The *SlMED18*-RNAi lines exhibited multiple developmental defects, including smaller size and slower growth rate of plant and significantly smaller compound leaves. The contents of endogenous bioactive GA_3_ in *SlMED18* silenced lines were slightly less than that in wild type. Furthermore, qRT-PCR analysis indicated that expression of gibberellins biosynthesis genes such as *SlGACPS* and *SlGA20x2*, auxin transport genes (*PIN1*, *PIN4*, *LAX1* and *LAX2*) and several key regulators, *KNOX1*, *KNOX2*, *PHAN* and *LANCEOLATE*(*LA*), which involved in the leaf morphogenesis were significantly down-regulated in *SlMED18*-RNAi lines. These results illustrated that *SlMED18* plays an essential role in regulating plant internode elongation and leaf expansion in tomato plants and it acts as a key positive regulator of gibberellins biosynthesis and signal transduction as well as auxin proper transport signalling. These findings are the basis for understanding the function of the individual Mediator subunits in tomato.

## Introduction

Plant growth and development, including vegetative growth and reproductive growth, is a multiphase process that requires a tight coordination among molecular, biochemical and structural elements^1^. The vegetative growth includes the development of root, stem and leaf, the reproductive growth includes flower, fruit and seed development and the vegetative growth is necessary for providing the essential nutrients for the reproductive growth. Plants usually grow vegetatively at early developmental stage then translate to the reproductive growth stage^2^. In the vegetative growth, the roots provide water and other nutrients to the plant, and the growth of the stem provides the possibility for the plant to produce more leaves, while the development and growth of the leaves provide more nutrients and energy for the reproductive growth of the plant.

The regulation of plant gene expression is necessary for the normal growth and development of plants, which is a complex and accurate network system that generally includes both transcription and translation of a series of genes. Transcription in eukaryotic organism is an intricate and extremely orchestrated process, through RNA Pol II assisted by a number of transcriptional regulators. The transcriptional regulators include transcriptional activators, various general transcription factors (GTFs), and a series of transcription cofactors^3,4^. Several transcription factor families, including MADS-box, GRAS, and MYB have been characterized for their regulatory roles in plant vegetative development^5–7^. In addition, Mediator, a multi-subunit complex, is one cofactor that promotes transcription initiation as the bridges between transcription activators bounding to regulatory upstream promoter or enhances DNA elements and the RNA Pol II general transcriptional machinery at the essential promoter^8–11^. Nowadays, Mediator complex has emerged as possibly the most essential section for regulating Pol II transcription in eukaryotes.

Mediator complex was first identified in *Saccharomyces cerevisiae* and structural studies showed that the 21 yeast Mediator subunits have been divided into four submodules, the head, middle, tail, and kinase modules^12^. Moreover, the modular architecture and subunit composition of Mediator complex is evolutionarily conserved in eukaryotes^13^. In plants, more than 30 different Mediator subunits have been shown that are part of the Mediator complex in different organisms. In the past, the studies of Mediator complex have been focused on yeasts and metazoans. Until 2007, more than a decade after its discovery in *S*. *cerevisiae*, Mediator complex was successfully purified from plants^14^. Since then, some plants Mediators and the physiological functions of several subunits began to be revealed. The discovery of the Mediator complex in *Arabidopsis thaliana* indicated the Mediator complex subunits possess their own specific function within the whole complex, and already, a majority of Mediator complex subunits have been reported to have important regulatory roles. In *Arabidopsis thaliana*, *AtMED12* and *AtMED13*, referred to as *GRAND CENTRAL* (*GCT*) and *CENTER CITY* (*CCT*), effect various aspects of plant development including embryo pattern formation, developmental timing and flowering and floral morphogenesis^15,16^. The genetic and physiological study suggested that *AtMED25*/ *PHYTOCHROME AND FLOWERING TIME 1* (*PFT1*) are implicated in regulating JA-triggered gene expression and effect flowering under suboptimal light conditions^14,17–21^. *AtMED14*, which was originally described as *STRUWWELPETER* (*SW*P), affects cell proliferation and shoot meristem development. *AtMED20a*, *AtMED18*, *AtMED8*, and *AtMED17* were known to implicated in non-coding RNA production^22,23^. *AtMED14*, *AtMED15*, and *AtMED16* have recently been speculated to be involved in defense signalling^24–26^. In another study, Arabidopsis *Med32* played a role in root development and senescence. Although there are some studies on the Mediator function in model plant which made some meaningful progress, the roles of plant mediator in RNA transcription remain to be further elucidated on account of the lack of relevant mutant gene material.

*MED18* (Mediator subunit 18 gene) is one of the Mediator complex subunits genes. Structural studies illustrated that *MED18* is located in the movable jaw of the head module structure and resembles the head of a crocodile with one limb^13^. Recently several studies about *MED18* in *Arabidopsis thaliana* have been reported that *AtMED18* was involved in regulating multiple plant physiological processes, including plant immunity, abscisic acid (ABA) responses, flowering time and floral organ identity through interactions with distinct transcription factors^27–30^.

*Solanum lycopersicum* is a kind of widely grown economic crop, which not only has high agricultural economic value but also is an important model plant for studying plants development. To date, although Mediator complex plays a critical role in promoting the transcription of genes, little is known about them in tomato. In this study, a member of mediator family, named Mediator subunit 18 (*SlMED18*), was isolated form tomato. To further explore the function of *SlMED18* in plant growth and development, we created transgenic tomato plants using S. lycopersicon Mill. cv. Ailsa Craig^++^ as wild type tomato by RNA interference. Down-regulation of *SlMED18* restricted internode elongation and leaf expansion, producing dwarf plants and smaller leaves than wild type. The molecular and cellular levels investigation of *SlMED18*-RNAi lines indicated that *SlMED18* plays an important role in regulating the development of leaf and stem. This research enhanced our knowledge about the roles of *SlMED18* in tomato developmental processes.

## Results

### ***SlMED18*** isolation and expression pattern analysis

In this study, a *Solanum lycopersicum* Mediator of RNA polymerase II transcription subunit 18 gene, named *SlMED18*, was isolated from wild type tomato leaves with specific primers (Supplementary Table S1) based on a cDNA clone (GenBank accession no. XM_010323502.2). Sequencing validation indicated that the correct gene sequence was obtained and sequence analysis showed that *SlMED18* contains an open reading frame (ORF) of 651 bp and encodes 216 amino acid residues.

**Figure 1:**
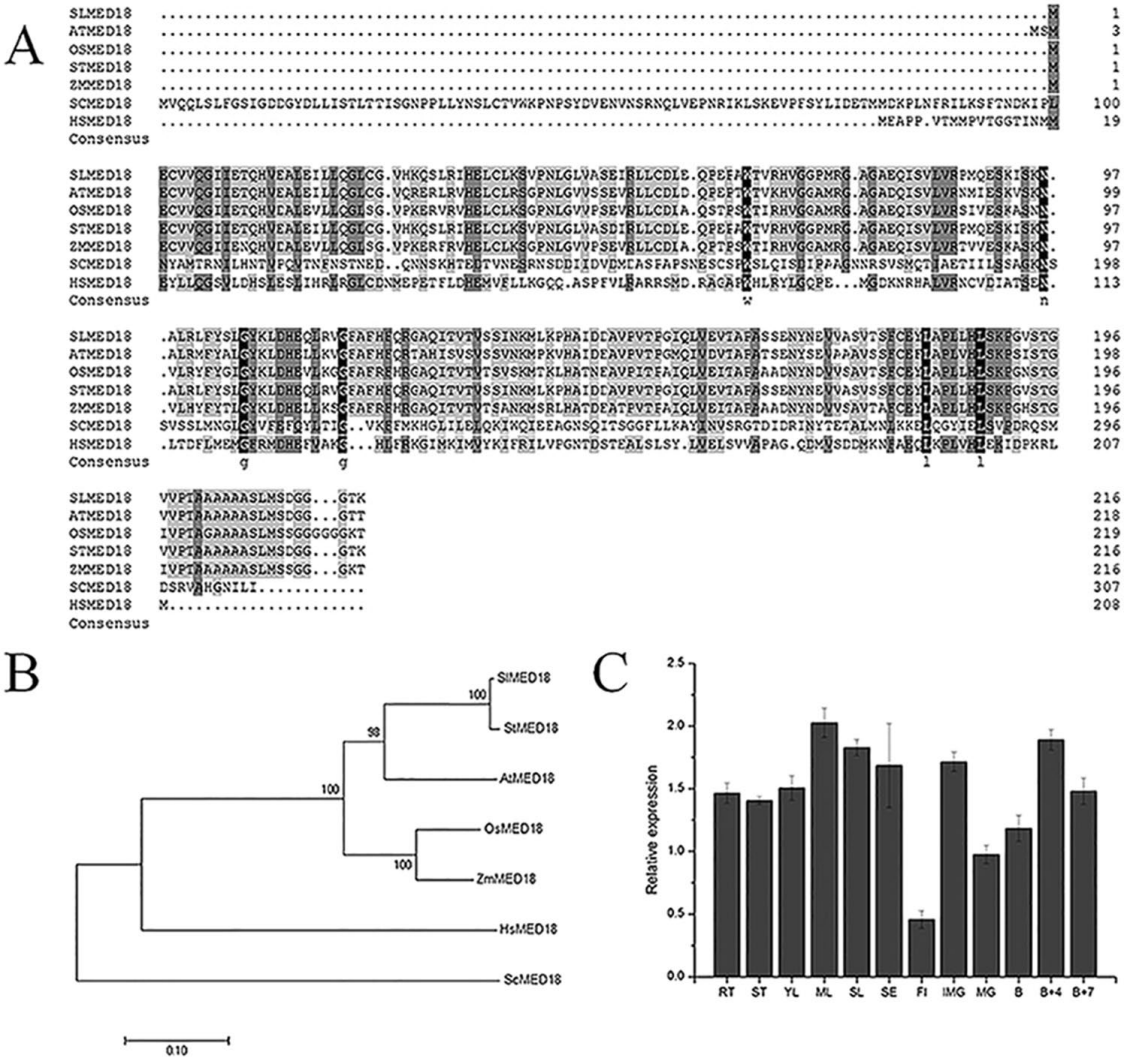
Evolutionary relationships and transcriptional pattern of *SlMED18* in wild type tomato. (**A**) Multiple sequence alignment of *MED18* protein in *S*. *lycopersicum* (Sl), *Arabidopsis thaliana* (At), *Oryza sativa* (Os), *Zea mays* (Zm), *Solanum tuberosum* (St), *S*. *cerevisiae* (Sc) and *Human* (Hs). The numbers on the right indicate the positions of amino acid residues. The identical amino acids are shaded in black, and similar amino acids are shaded in gray. (**B**) Phylogenetic relationship of *MED18* in tomato and other species. The tree was constructed from a complete alignment of 7 *MED18* amino acid sequences by MEGA7, using the Neighbor-Joining method. And the evolutionary distances were computed by the p-distance method. (**C**) Transcriptional pattern of *SlMED18* in wild type tomato. RT, root; ST stem; YL, young leaf; ML mature leaf; SL, senescent leaf; SE, sepal of flower in anthesis; FI, flower; IMG, immature green fruit; MG, mature green fruit; B, breaker fruit; B + 4, 4 days after breaker fruit; B + 7, 7 days after breaker fruit. Each value represents the mean ± SD of three replicates.

In addition, keyword searches and BLAST searches were performed against the *Arabidopsis thaliana* Mediator complex subunit gene sequences. To dissect the evolutionary relationships of *MED18*, the *MED18* sequences of tomato, *Zea mays*(Zm), *Solanum tuberosum* (St), *S*. *cerevisiae*, *Human* (Hs), the dicot model plant *Arabidopsis thaliana* (At) and the monocot model plant *Oryza sativa* (Os)were used to conduct multiple sequence alignment and an original tree was constructed. Phylogenic analysis implied that sequences are weakly conserved between *S*. *cerevisiae*, *Human* (Hs) and plants, but they are conserved across the plant kingdom (Fig. 1A,B).

As we all know that the tissue specificity of gene expression may be correlated with specific biological functions. So the real-time PCR analysis was conducted to clarify the expression profile of *SlMED18* in various tissues, which consist of roots, stems, leaves, flowers and fruits at different developmental stages (Fig. 1C). The results showed that *SlMED18* expressed abundantly in all the organizations we examined, which was consistent with pre-expression pattern (Supplementary Fig. S1). The bar graph of expression profile in various tomato tissues was obtained from Tomato lab website. These results indicated that *SlMED18* may have essential roles in multiple tomato plant growth and development process.

### Repression of SlMED18 causes plant developmental defects

**Figure 2:**
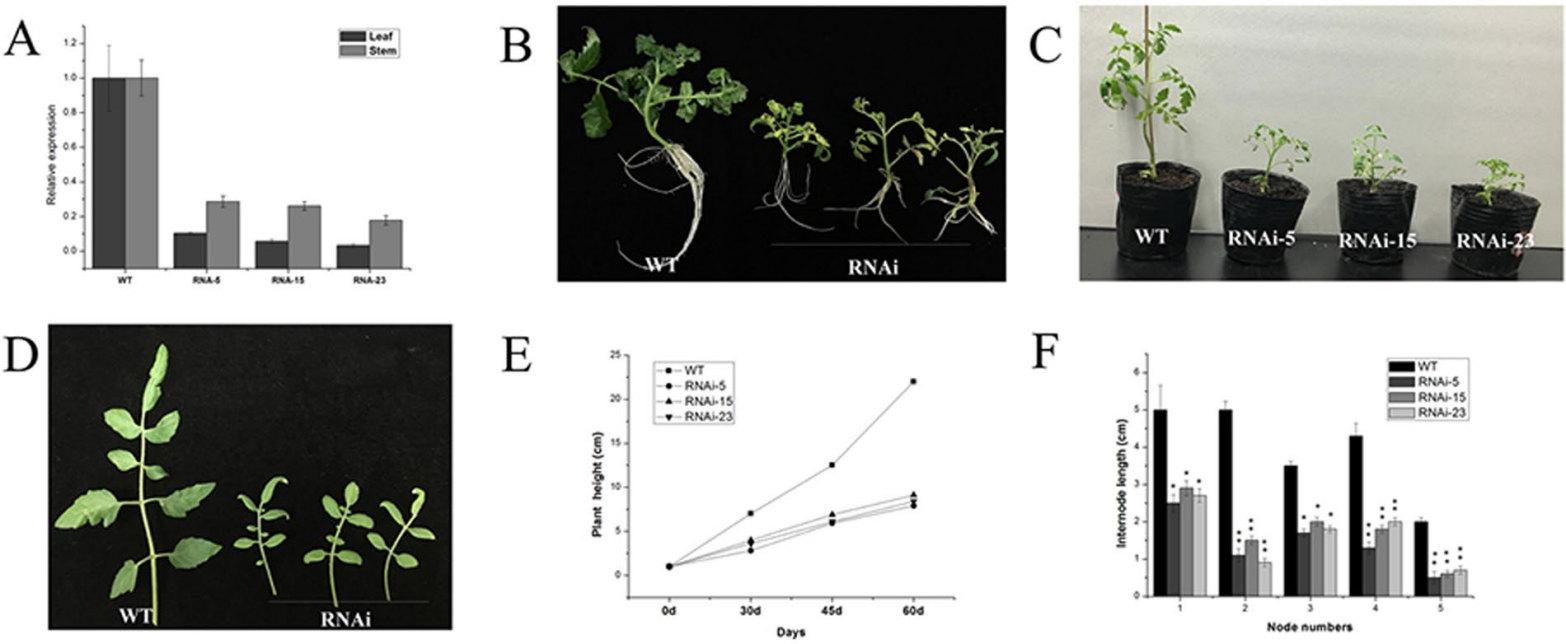
Repression of SlMED18 causes plant developmental defects. (**A**) The expression levels of *SlMED18* in three *SlMED18-*RNAi transgenic lines (RNAi-5, RNAi-15 and RNAi-23) and wild type plants. We collected young leaves and stems of the plants as materials detected by real-time qPCR and normalized the expression data of wild type plants to 1. (**B**,**C**) The *SlMED18*-RNAi plants (RNAi-5, RNAi-15 and RNAi-23) were exhibited multiple developmental defects. 30-day old plants rooted on the MS culture medium in culture bottle (**B**) and then transplanted them into pots to cultivate 30 days (**C**). (**D**) Phenotypes of leaves in *SlMED18*-RNAi lines. The compound leaves were collected from 60-day-old wild type and *SlMED18*-RNAi lines at the same nodes. (**E**) The growth rate of wild type and *SlMED18*-RNAi plants. We measured the height of plants in 0-day old, 30-day old, 45-day old and 60 day old. (**F**) Internode length of the first to the fifth node from 60-days-old wild type and *SlMED18*-RNAi lines.

**Figure 3:**
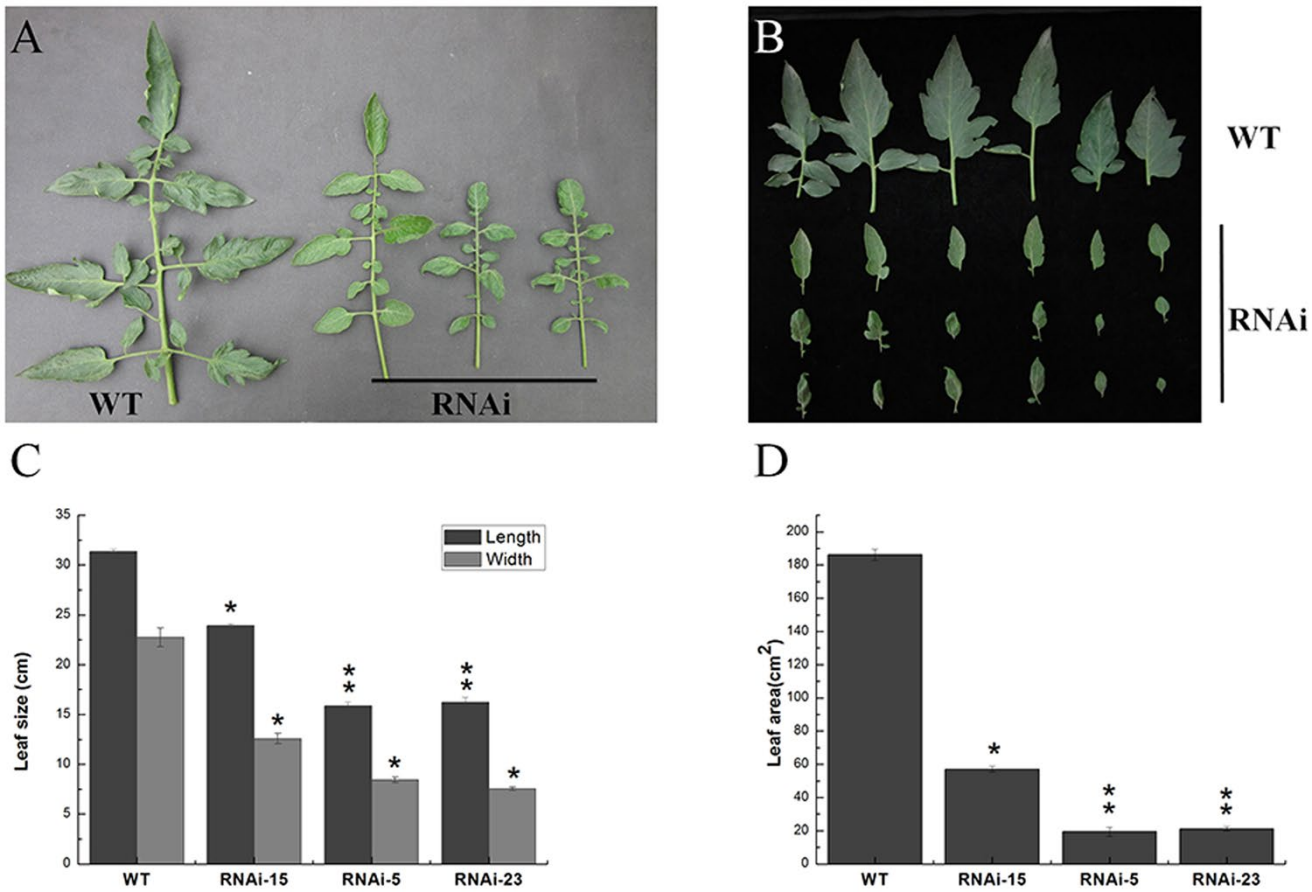
Altered leaf morphology in *SlMED18*-RNAi tomato plants. The compound leaves (**A**) and the main leaflets (**B**) were collected from the same node (the fifth node) of 60-days old wild type and *SlMED18*-RNAi tomato plants. The maximum length, maximum width (**C**), and the area of leaves (**D**) compered between wild type and *SlMED18*-RNAi tomato plants. Error bar indicates means ± SD (n = 8). *P < 0.05 and **P < 0.01 according to *t*-test.

To further investigate the biological functions of *SlMED18* in tomato, an RNAi expression vector targeting *SlMED18* gene was created and transformed into tomato (Solanum lycopersicum ‘Ailsa Craig’ AC^++^) via Agrobacterium tumefactions mediated transformation. 25 independent transgenic lines were confirmed by PCR using primers of *NPT II* (Supplementary Table S1), then their total RNAs were extracted from young leaves to investigate the relative expression of *SlMED18*, respectively. qRT- PCR data showed that the three transgenic lines (RNAi 5, RNAi 15 and RNAi 23), compared with the control, displayed 90%, 93% and 96% reduction in *SlMED18* mRNA levels, respectively (Fig. 2A). Later these three transgenic lines were chosen and used for subsequent experiments. Compared with wild type plants, the *SlMED18*-RNAi lines displayed shorter and thinner stem with significantly smaller compound leaf (Fig. 2B–D and Supplementary Fig. S2A–C). The height of 60-days-old wild type plant was about 22.5 cm, while the three transgenic lines (RNAi 5, RNAi 15 and RNAi 23) exhibited a lower growth rate and had 9.1, 8.8 and 8.0 cm plant height. Thus, the RNAi 5, RNAi 15 and RNAi 23 plants were 59.56%, 60.9% and 64.34% shorter than the control, respectively (Fig. 2E). Furthermore, the internode lengths of 60-days-old wild type and *SlMED18*-RNAi plant from the first to the fifth node were measured, the result showed that every corresponding internode in *SlMED18*-RNAi transgenic tomato plants was reduced remarkably compared with the wild type (Fig. 2F). Morphology observation displayed that the stems of *SlMED18*-RNAi lines were thinner than the wild type (Fig. S2A,C), and the leaves were significantly smaller (Fig. 3A,B). The length, width as well as area of mature compound leaves was significantly less than wild type (Fig. 3C,D).

### Transgenic plants have smaller leaf cells

**Figure 4:**
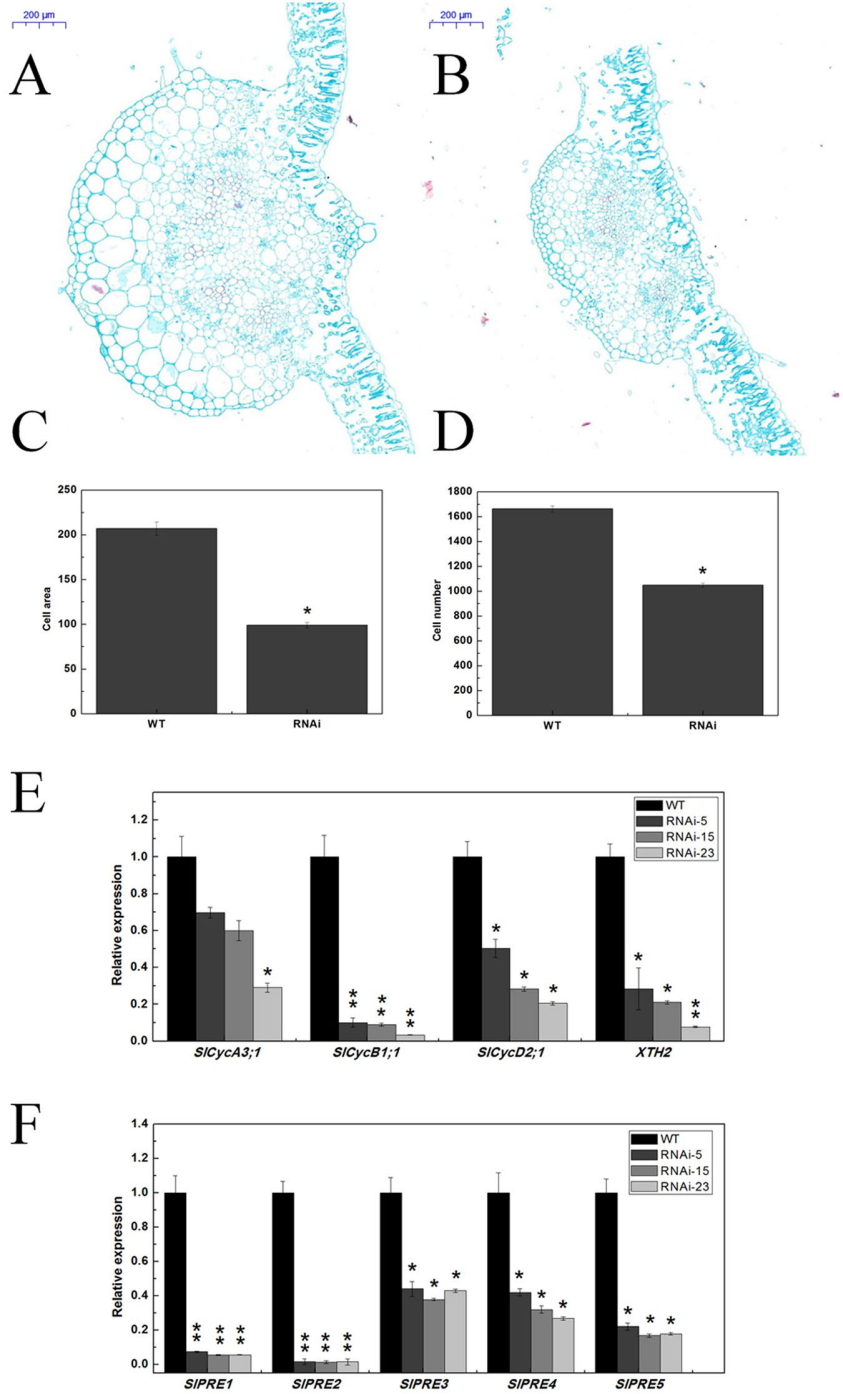
Anatomical analyses of leaf between wild-type and the *Sl*MED18-RNAi lines. (**A** and **B**) The lead of wild type (**A**) and *SMED18*-RNAi plant (**B**) were prepared transverse sections. Bars = 200 μm. (**C** and **D**) The estimated cell area (μm^2^) (**C**) and cell number of leaf vein (**D**) were measured and compared between transgenic and wild type at the same position in both cases. Data are shown as the mean ± SE. (**E** and **F**) Expression of several genes associated with cell expansion and cell cycle genes (**E**) *SlCycA3;1*, *SlCycB1;1*, *SlCycD2;1*, *SlXTH2*; (**F**) *SlPRE1*, *SlPRE2*, *SlPRE3*, *SlPRE4* in leaf.

Given the differences of leaves between transgenic lines and the wild type, an anatomical investigation of sections of leaf was performed to investigate whether this phenotype was associated with a decrease in the cell size or in the cell number. The cellular analysis showed that the size of leaf cell in the *SlMED18-*RNAi lines was dissimilar from those in the wild type at the same stage of tomato plant development (Fig. 4A–B). The cellular data indicate that cell size of the *SlMED18*-RNAi leaf was significantly smaller and the cell number of leaf vein was notably less, compared with those in the wild type (Fig. 4C and D). The results suggested that the smaller and narrower leaves might due to both the reduction of leaves cell size and cell number.

To explore the molecular mechanism of smaller leaf of transgenic plants, some genes associated with cell expansion and cell cycle genes, such as *SlCycA3;1*, *SlCycB1;1*, *SlCycD2;1*, *SlXTH2*, *SlPRE1*, *SlPRE2*, *SlPRE3*, *SlPRE4* and *SlPRE5* were examined in leaf. As shown in Fig. 4E, the representative cell cycle regulatory genes, *SlCycA3;1*, *SlCycB1;1* and *SlCycD2;1*^31^, and the biogenesis and modification of the cell wall components related genes, *SlXTH2*^32^, were down-regulated in *SlMED18*-RNAi lines. Moreover, the transcript levels of *SlPRE1*, *SlPRE2*, *SlPRE3*, *SlPRE4* and *SlPRE5*, which involved in cell division and expansion^33^, were markedly decreased in *SlMED18*-RNAi lines (Fig. 4F).

### *SlMED18*-RNAi plants have decreased endogenous bioactive Gibberellins (GAs) contents

**Figure 5:**
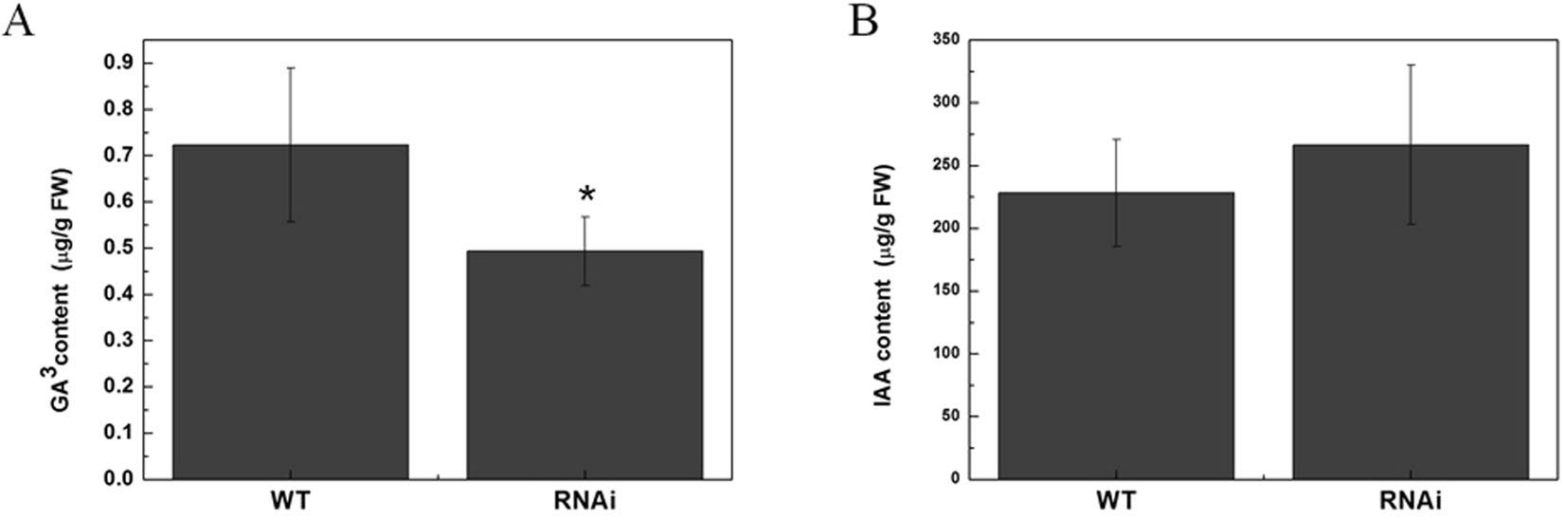
The concentration of endogenous gibberellin GA_3_ (**A**) and IAA (**B**) in apical shoots stem of wild type and transgenic lines. We used three wild type simples and three transgenic simples to do biological duplication and the value stand for the mean ± SD of three replicates.

**Figure 6:**
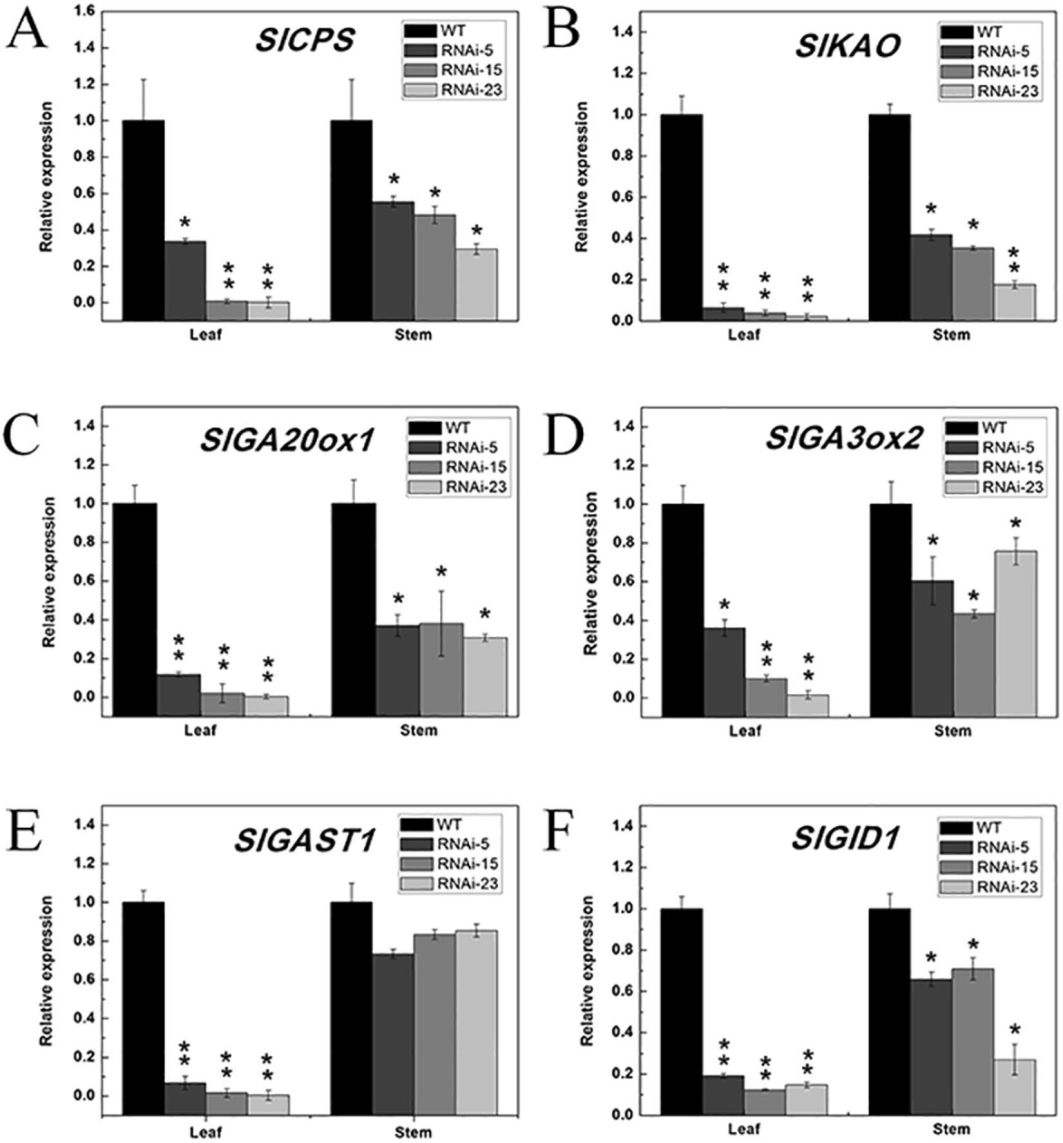
Genes involved in GAs biosynthesis and signal transduction in *SlMED18-*RNAi plants. (**A**–**F**) Show the expression levels of *SlCPS*, *SlKAO*, *SlFA20ox1*, *SlGA3ox2*, *SlGAST1* and *SlGID1* in wild-type and transgenic lines. The relative expression of each gene in the wild-type was set to 1.0 and the Error bar indicates the mean ± SD of three replicates (n = 8). *P < 0.05 and **P < 0.01 according to t-test.

It has been reported that the plant hormone GAs is involved in internode elongation as well as other plant developmental processes. To date, a lot of genes encoding GAs biosynthetic or signalling transduction genes have been characterized as being associated with this process^34,35^. As the *SlMED18*-RNAi plants were dramatically shorter than the wild type (Fig. 2C), the contents of endogenous bioactive GA_3_ in the stems of wild type and *SlMED18*-RNAi lines were measured. The results showed that endogenous GA_3_ in transgenic plants were significant less than that of wild type (Fig. 5A), indicating that the dwarfism of *SlMED18*-RNAi plants is due to the deficiency of endogenous GAs. Therefore, we further detected some genes involved in the GAs biosynthesis and signal transduction. The transcript accumulation of two genes, tomato ent-copalyl diphosphate synthase (*SlCPS*) and tomato ent-kaurenoic acid oxidase (*SlKAO*), which involved in the GA biosynthetic pathway were markedly reduced in stems and leaves of *SlMED18*-RNAi lines (Fig. 6A,B). In many plant species, *GA20oxs* are the core GA biosynthetic enzymes that determine the GAs concentration and *GA3oxs* catalyze the final step to produce bioactive GAs (GA1, GA3, and GA4)^36^. In our study, *SlGA20ox1* and *SlGA3ox2* were dramatically down-regulated in leaves and stems of *SlMED18*-RNAi lines compared with the wild type (Fig. 6C,D). To further assess whether *SlMED18* affects GA signal transduction factors, we also determined the expression level of *SlGAST1* (tomato gibberellin-stimulated transcripts 1), a tomato GA-responsive downstream gene, as well as a GA receptor *SlGID1-A* (tomato GA INSENSITIVE DWARF1-A)^37–39^. The results displayed that *SlGAST1* and *SlGID1-A* were significantly down-regulated in leaves of *SlMED18*-RNAi plants but not drastically decreased in stems of transgenic plants (Fig. 6E,F). These results denoted that *SlMED18* affects GA biosynthesis and signal transduction.

### Silencing of *SlMED18* alters the expression of auxin transport and response genes

**Figure 7:**
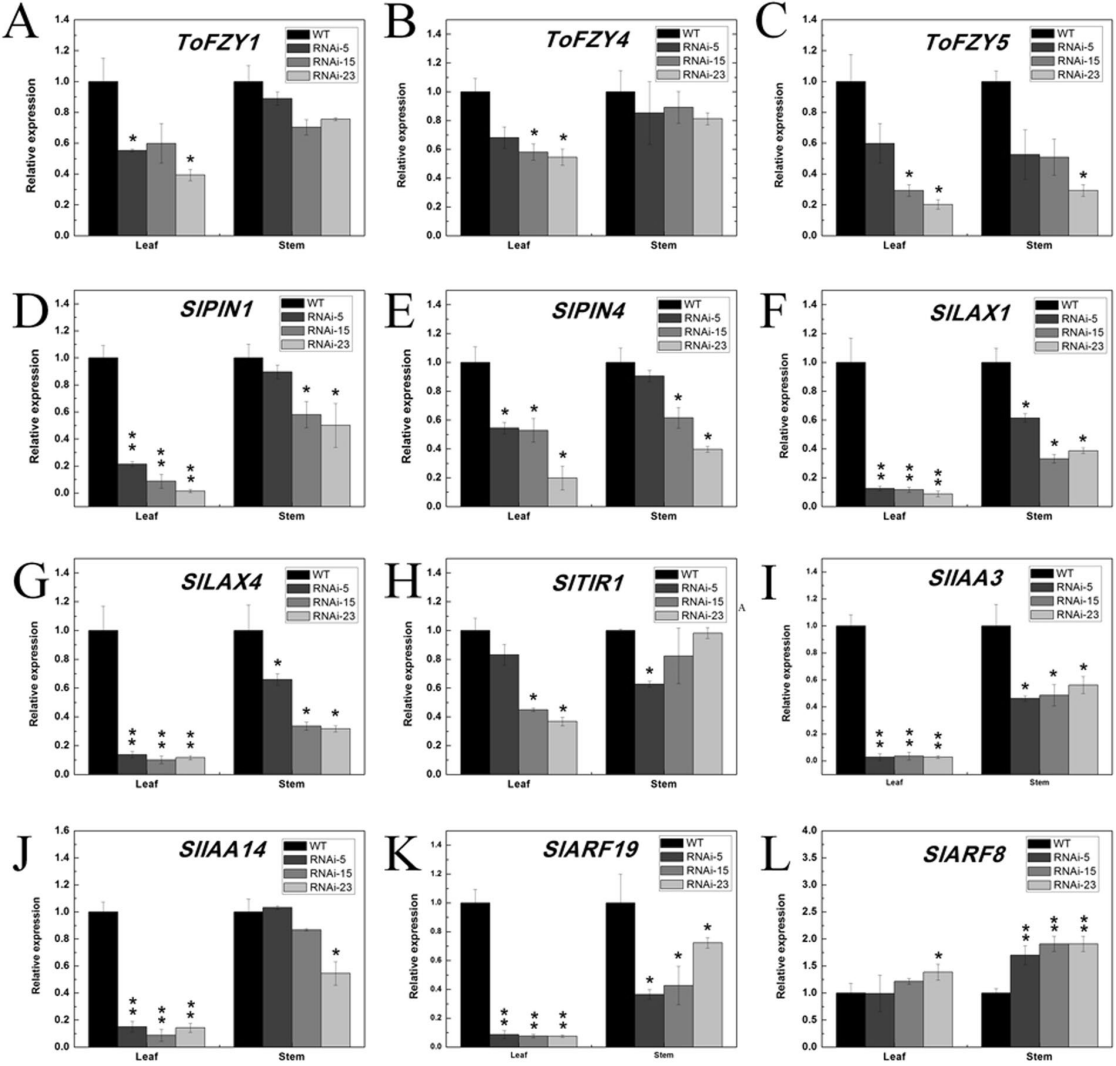
(**A**–**C**) The expression levels of three genes ToFZY1, ToFZY4 and ToFZY5, which contributed to the localized biosynthesis of IAA. (**D**–**G**) Expression of several key genes of auxin transport and auxin-responsive in *SlMED18*-RNAi transgenic lines. The relative mRNA level of major polar auxin transport genes *SlPINI* (**D**), *SlPIN4* (**E**), *SlLAX1* (**F**) and *SlLAX4* (**G**) in leaf and stem, respectively. (**H**) Transcript accumulation of *TIR1*, an auxin receptor. (**I**–**L**) The auxin-responsive genes (*SlIAA3*, *SlIAA14*, *SlARF19* and *SlARF8*) in leaf and stem tissues were investigated by qRT-PCR. The expression in each sample was used for standardization, and the relative expression of each gene in the wild-type was set to 1.0. Each value represents the mean ± SD of three replicates (n = 8). Asterisks indicate a significant difference (*P < 0.05 and **P < 0.01) between wild type and transgenic lines, according to t-test.

In our study, we compare the expression level of three genes *ToFZY1*, *ToFZY4* and *ToFZY5*, which contributed to the localized biosynthesis of IAA, between *SlMED18*-RNAi plants and wild type^40^. In transgenic plant, *ToFZY1*, *ToFZY4* and *ToFZY5* were down-regulated. However, the contents of IAA in the stems of *SlMED18*-RNAi lines showed no significant change compared to wild type (Fig. 5B). The spatiotemporal localization of auxin acts as an essential regulator of leaves organogenesis and stems growth. The polar auxin transport mediated by PIN and AUX/LAX proteins is a major mechanism that regulates auxin distribution, which controls cellular auxin efflux and influx respectively^41^. So we further detected the expression of auxin transport and response genes in *SlMED18*-RNAi plants and wild type. In this study, two PIN genes, *PIN1* and *PIN4*, and two LAX genes, *LAX1* and *LAX2*, were examined in stems and leaves of the transgenic lines and the wild type, respectively. The results demonstrated that these four genes were significantly down-regulated in the stems and leaves of the transgenic lines (Fig. 7D–G). *TIR1* is reported to be an auxin receptor that mediates rapid degradation of Aux/IAA proteins and consequently changes the expression of auxin-regulated genes^42^. In our study, the expression level of *SlT1R1* was markedly decreased in leaves of transgenic lines but no significant changes were observed in stems of the transgenic lines compared with the wild type (Fig. 7H). These results may explain the no significant change of IAA content that due to the severe inhibition of auxin transport and the IAA were accumulated at the apices of the stem. We also examined the expression of two genes of Aux/IAA family, *SlIAA3* and *SlIAA14*, which are known to be implicated in modified auxin response. The results displayed that the transcripts of *SlIAA3* were remarkably reduced in leaves and stems of transgenic lines and *SlIAA14* was significantly down-regulated in leaves but not in stems of transgenic lines (Fig. 7I,J). *SlARF19*, a transcriptional activator of early auxin response gene, was also significantly reduced in *SlMED18*-RNAi lines (Fig. 7K).The relative expression level of auxin response gene *SlARF8*, which may control the free IAA level in a negative feedback fashion^43^, was up-regulated in the *SlMED18*-RNAi lines compared with wild type (Fig. 7L). As a result, our study suggested that repression of *SlMED18* may affect auxin regulation by regulating the expression of polar auxin transport genes and auxin responsive genes.

### Suppression of *SlMED18* results in down-regulated expression of leaf morphogenesis related genes

**Figure 8:**
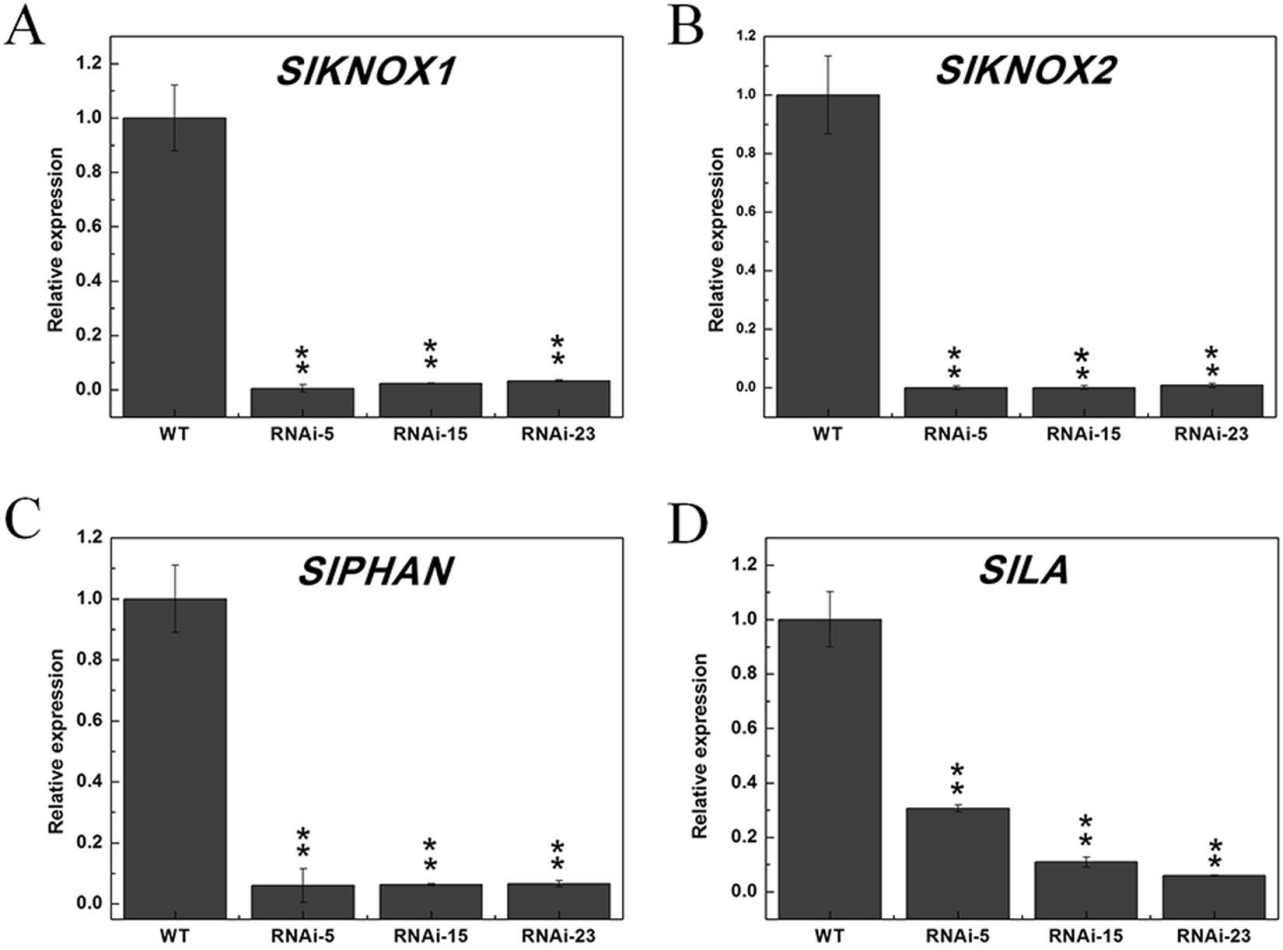
qPCR analysis of key regulators involved in the leaf morphogenesis. The relative expression of *SlKNOX1* (**A**), *SlKNOX2* (**B**), *SlPHAN* (**C**) and *SlLA* (**D**) showed various changes. The error bar indicates the mean ± SD of three replicates. Asterisks indicate a significant difference (*P < 0.05 and **P < 0.01) as determined by student’s t-test.

Given that the *SlMED18*-RNAi transgenic lines had significantly smaller compound leaves with narrower lamina compared with wild type lines. (Fig. 3A and B), we detected the expression of four genes, *KNOX1*, *KNOX2*, *PHAN* and *LANCEOLATE* (*LA*), which are associated with leaf morphology and development by qRT-PCR in the leaves of 90-day old wild type and *SlMED18*-RNAi plants. The results showed that the transcription levels of these genes were sharply decreased in the *SlMED18*-RNAi lines compared with the wild type (Fig. 8A–D). These results suggested that *SlMED18* may affect leaf morphology development through regulating the expression of these key regulators.

## Discussion

Mediator complex is a central part of the transcriptional machinery in all eukaryotes. Since the first purification of Mediator complex from yeast, subsequent studies of yeast Mediator subunit mutants expression profiling exposed that some specific sets of genes can be directly regulated by Mediator^44^. In plants, the research of Mediator is not lagging so far. Over the past few years, a series of reports have emerged revealing the Mediator subunits are essential in regulating diverse processes like embryo pattern formation, developmental timing, flowering and response to multiple different biotic and abiotic stresses^15,45,46^. However, the information about function of the Mediator subunits on plant developmental processes is lacking and the studies on Mediator subunits among plant kingdom only focus on *Arabidopsis thaliana* and *Oryza sativa*. To date, there is little research about the function of Mediator in tomato, which is one of the important model plants.

*MED18* is part of the head module of Mediator complex, and some research identified that *MED18* plays essential roles in multiple biological functions, including plant responses to microbial infection and regulating flowering and germination by environmental signals in *Arabidopsis*^28,29^. Here, phylogenetic analysis among *MED18* in tomato, *S*. *cerevisiae* (*Sc*), *Human* (*Hs*) and several typical model plants showed that Mediators despite have low sequence homology between *S*. *cerevisiae* (*Sc*), *Human* (*Hs*) and plants, but they conserved across plant kingdoms (Fig. 1A,B). In addition, the *MED18* was isolated from tomato and expression profile exhibited that *SlMED18* was extensively expressed in all of the examined tissues, illustrating the *SlMED18* may have effect on different plant physiological processes. Unlike the report in Arabidopsis mutant^29^, silencing of *SlMED18* in tomato caused severe plant developmental defects, including smaller size and slower growth rate of the plant with significantly smaller compound leaves and narrower lamina and smooth margins (Fig. 2B–D and Supplementary Fig. S2A–C). The result suggested that *SlMED18* plays important roles in the plant developmental processes.

In *SlMED18-*RNAi lines, the plant height, width and length of compound leaves and leaf area were measured. The date explicated that the stem and leaf developmental result in a strong reduction in plant growth of whole plant (Figs 2E,F and 3C,D). Moreover, the anatomical characterization of leaf from *SlMED18* silenced plants and wild type was performed and we found that the size and number of leaf cell in *SlMED18-*RNAi lines were drastically decreased (Fig. 4C,D). The genes associated with cell expansion and cell cycle genes, including *SlCycA3;1*, *SlCycB1;1*, *SlCycD2;1*, *SlXTH2*, *SlPRE1*, *SlPRE2*, *SlPRE3*, *SlPRE4* and *SlPRE5* (Fig. 4E,F) were remarkable up-regulated in *SlMED18*-RNAi lines. These results may partially explain the smaller compound leaves in the transgenic lines.

Plant hormones play the crucial roles in regulating plant growth and development as well as plant responses to various biotic and abiotic stress responses. Among the seven different kinds of plant hormones, gibberellin (GAs) and auxin are both key signals in plant developmental and often act synergistically. Some research of dwarf mutants and analysis of their GAs contents made sure that bioactive GAs is associated with the regulation of stem growth in plants^47^. In tomato, gibberellin-responsive mutants (gib-l, gib-2, and gib-3) were identified by reduced plant height due to shorter internodes and their leaves are smaller, darker green, and structure differently as compared to wild type^48^. In our study, the endogenous GA_3_ content of transgenic lines is less than wild type (Fig. 5), demonstrating that the dwarf plant and smaller leaves phenotype of *SlMED18-*RANi lines may be ascribed to the decreased levels of endogenous GAs. As we all know, the content of endogenous GAs is regulated by the expression of several key genes involved in GAs biosynthesis and signal transduction such as *CPS*, *KAO*, *SlGA20oxs* and *SlGA3ox*. In previous reports, a number of examples explicated that these genes were involved in alteration of GAs level and lead to a developmental defect plant. Loss-of-function CPS mutants of several plant species, such as *Oryza sativa* and *Arabidopsis thaliana*, resulted in severely impaired plant growth^49,50^. In *Arabidopsis thaliana*, kao1 kao2 double mutant shows typical non-germinating GA-dwarf phenotypes^51^. Besides, in tomato, up-regulation of *SlGA20oxs*, and *SlGA3ox* can lead to higher levels of bioactive GAs and constitutive co-suppression of the *SlGA20ox1* gene cause serious defects in vegetative and reproductive development. In our study, the expression of *SlCPS*, *SlKAO*, *SlGA20ox1* and *SlGA3ox2* were dramatically decreased in leaves and stems of SlMED18-RNAi lines (Fig. 6A–D), demonstrating that repression of *SlMED18* lead to severe plant developmental defects by down-regulating the expression of key GA biosynthesis enzyme genes. However, biosynthesis of endogenous bioactive gibberellin (GA) is a complicated process, including the regulated at transcription level, the post-transcriptional level, translation level and post-translational level of key GA biosynthesis enzyme genes. Especially, in eukaryotic, many proteins undergo extensive post-translational modifications such as methylation, acetylation, phosphorylation, glycosylation, and ubiquitination can affect the protein content. For example, ubiquitination has been shown to be involved in the regulation of protein degradation and gene expression. It may explain the reduction in gibberellins contents present in the transgenic line compared with the wild type was not as significant as the expression levels of GA related genes. In addition, the expression levels of a GA response gene *SlGAST1*^37^ and *SlGID1-A* were also notably reduced in the leaves of *SlMED18*-silenced lines but not significantly changed between the wild type and *SlMED18*-RNAi stems (Fig. 6E,F), probably because of the feedback of GA deficiency.

The plant hormone auxin has a key role in many developmental processes, and it is known that the level of auxin throughout whole plant tissues is tightly controlled the coordination of plant growth and development^52^. The multidimensional effects of auxin need to coordinate regulation of auxin influx and efflux carriers, guiding the auxin transport in a polar way that together form a polar auxin transport (PAT) network^53^. Unlike other acknowledged plant hormones, auxin can be actively transported in a directional fashion in order to affect auxin concentrations during phototropic and gravitropic response. Two largest participators of directional transport in the PAT system are the PIN-FORMED (PIN) auxin transporters, which act as auxin efflux carriers, and the AUX1 (AUXIN RESISTANT1) and its close homologues LAX (LIKE AUXIN RESISTANT) that take participate in auxin influx as the influx carrier. The *PIN1*, *PIN4*, *LAX1*, *LAX4* genes were dramatically declined (Fig. 7D–G) and several auxin responsive genes were also changed in *SlMED18* RNAi lines (Fig. 7H–L). The results collectively suggested that *MED18* takes part in the regulation of auxin distribution, possibly through controlling the activity of polar auxin transport genes.

Gibberellin (GA) and auxin are both key signals in plant growth and are often observed to act synergistically^54^. However, much of our knowledge on GAs and auxin has come from studying them independently. Recently, some studies proposed a new idea that there is crosstalk between GA signalling and auxin transport. There are some reports that the auxin can induce the transcription of several GA biosynthesis genes such as in Arabidopsis, after decapitation the apical source of auxins, the active GA levels in stems are lower than those in stems of intact plants^55^. In contrast, GA biosynthesis and signalling has a positive impact on proper auxin transport^56^. In Arabidopsis GA mutants, the GA pathway cross-talk with PIN protein–dependent auxin transport pathway lead to the auxin transport impairment in mutants, which causative for defection of cotyledon differentiation and root gravitropic responses^57^.

Several genes, including class 1 *KNOTTED* homeodomain genes (*KNOX*), *PHANTASTICA* gene *PHAN* and *CIN-TCP* transcription factor *LANCEOLATE* (*LA*), have been characterized for their regulatory roles in altering leaf shape and development. *PHAN* is required for normal meristem function^58^. The KNOX gene in tomato was involved in organ leaf formation, and the tomato *LANCEOLATE* (*LA)* gene was shown to promote leaf differentiation^59,60^. Silencing *SlMED18* in tomato altered the expression of these genes (Fig. 8A–D), suggesting that *SlMED18* regulates the leaf development. Interestingly, recently these genes were exhibited to act in part by adjusting gibberellin and auxin levels. KNOX proteins partly linked to GAs pathways and have a positive impact on auxin signalling^61^. It is reported that expression of the *SlGA20ox1* is up-regulated in gain-of-function La mutants, illustrating that *LA* activity is partly mediated by positive regulation of the GAs response, probably by regulation of endogenous bioactive GAs levels^59^. In addition, in leaf primordial of harboring the dominant La mutant the auxin signal is very weak, implying that auxin appear to affect the specification of marginal outgrowths, controlled by LA. Thus, narrower lamina of *SlMED18*-RNAi lines probably not only accounts for the alteration of the key genes for leaf development but also is induced by modulation of GA and auxin signalling. Additionally, we also found the repression of *SlMED18* altered inflorescences architecture (Supplementary Fig. A and B), whether this is due to the direct affection on transcriptional regulators or indirect result of hormone signalling pathways, future biochemical analyses will need to identify.

*S*ilencing *SlMED18* in tomato caused multiple plant developmental defects, including smaller size and slower growth rate of plant and significantly smaller compound leaves, further we found that *SlMED18* affected a set of transcription factors to regulate diverse physiological and cellular processes through impacting intra and extracellular signals. Furthermore, understanding the function of this Mediator subunit in tomato may provide information for entire tomato Mediator complexes.

## Materials and Methods

### Plant materials and growth conditions

In this article, We planted the tomato (*Solanum lycopersicum*, ‘Ailsa Craig’ AC^++^) as wild type that together with transgenic cultures were grown in controlled greenhouse conditions (16-hour-day (25 °C)/8-hour-night (18 °C)cycle, 80% humidity and a 250 μ mol m^−2^ s^−1^ light intensity) and managed them routinely. The sample of roots, stems, young leaves, mature leaves, senescent leaves, flowers, sepals and fruits of five periods were collected from tomato for *SlMED18* organ-specific expression profiling. Transgenic tomato plants that came from tissue culture in first generation (T0) were used in our experiment because the transgenic plants were sterility. For analysis of gene expression, the fifth leaf from top and the young stem that at the corresponding developmental stage from the 90-day-old plant were collected. Plant samples that we used were frozen in liquid nitrogen immediately then stored at −80 °C.

### Phylogenetic Analysis

The cDNA and protein sequence date of tomato mediator of RNA polymerase II transcription subunit 18 was found in NCBI (http://www.ncbi.nlm.nih.gov/) and Sol Genomics Network (SGN, http://solgenomics.net/). *MED18* in *Solanum lycopersicum* (Sl), *Arabidopsis thaliana* (At), *Oryza sativa* (Os), *Solanum tuberosum* (St), *Zea mays* (Zm), *Saccharomyces*. *cerevisiae* (Sc) and *Human* (Hs) were obtained from the NCBI databases. GenBank accession numbers for multiple sequence alignment and phylogenetic analysis are as follows: *SlMED18* (XP_010321804), *AtMED18* (NP_565534), *OsMED18* (XP_015625319), *StMED18* (XP_006364546), *ScMED18* (NP_011618) and *HsMED18* (NP_001120822). Multiple sequence alignment of *MED18* in tomato with other species was conducted by DNAMAN 6.0 programs. Alignments of amino acid sequences of *MED18* subunits proteins were made using Clustal X2.1^62^. A phylogenetic tree of Mediator of RNA polymerase II transcription subunit 18 protein sequence in 7 different species was constructed using MEGA7 (http://megasoftware.net/) and the phylogenic analysis was inferred using the Neighbor-Joining method.

### Total RNA extraction and quantitative real-time PCR analysis

Using RNAiso Plus (Takara) in accordance with the instructions, we extracted Total RNA from wild type and MED18-RNAi plant tissues. cDNA was synthesized with oligo(dT)_20_ as primer by RNA that reverse-transcribed using M-MLV reverse transcriptase (Promega). In addition, the synthesized cDNA need to fold dilute three times with nuclease-free water for quantitative real-time PCR analysis. Additionally, the qRT-PCR analysis was conducted with the GoTaq qPCR Master Mix (Promega), 1.0 μL mixture primers, 1.0 μL cDNA, 3.0 μL ddH_2_O by CFX96™ Real-Time System (Bio-Rad, USA). We performed the NRT (no reverse transcription control) and NTC (no template control) to eliminate the effect by genomic DNA and the environment factor. The organ-specific expression analysis was detected using the *SlCAC* gene (accession number: SGN-U314153) which showed stable expression across diverse tissues, as internal standard^38^. The 2^−ΔΔCT^ method was used to performed relative gene expression levels analysis^63^. In addition, each sample was repeated three times and standard curves were run at the same time. All primers we used were shown in Supplementary Table S1 that designed by Primer premier 6.24 software (http://www.premierbiosoft.com/crm/jsp/com/pbi/crm/clientside/ProductList.jsp).

### Expression analysis prediction of *SlMED18*

*SlMED18* expression prediction atlas of tomato tissues were obtained using Tomato lab website (http://tomatolab.cshl.edu/~lippmanlab2/allexp_query.html). In this website, the tomato genome sequence provided form Tomato Genome Consortium and searched by gene ID came from Sol Genomics Network (http://solgenomics.net/)^64,65^.

### *SlMED18*-RNAi vector construction and plant transformation

To generate *MED18*-RNAi lines, an RNAi vector was constructed. Primers of *SlMED18*-RNAi were showed in Supplementary Table S1, tailed with *XhoI*, *XbaI* and *HindIII*, *KpnI* restriction sites at the 5′end respectively, were used to amplify a 343-bp specific fragment of *SlMED18* cDNA. We used cloning vector pHANNIBAL as the original vector, digested the above amplified fragment of *SlMED18* products that with *HindIII*/*XbaI* and *KpnI*/*XhoI*, inserted into the pHANNIBAL plasmid at the *HindIII*/*XbaI* restriction site in the sense orientation while at the *KpnI*/*XhoI* restriction sited in the antisense orientation. Lastly, a double-stranded RNA expression unit, covered the calf mosaic virus (CaMV) 35 S promoter, was purified and then integrated into a plant binary vector pBIN19 with *SacI* and *XbaI* restriction sites. After *SlMED18*-RNAi Vector constructed, the generated binary plasmids, confirmed by restriction digest analysis and sequencing validation, were transferred into the *Agrobacterium tumefactions LBA4404* strain, then the *Agrobacterium tumefaciens*-mediated transformation was carried out and the *SlMED18*-RNAi lines were obtained^5^. The transgenic plants rooted on MS solid medium containing kanamycin for selecting the positive transgenic lines and the primers NPTII-F/-R were used to detect the *MED18*-RNAi plants (Supplementary Table S1) and the positive transgenic lines were used for further study.

### Quantification of phenotypes and statistical analysis

To study the differences between the transgenic plants and wild type, in addition to the height and internode length were measured; the length, width and area of mature leaf (4–5 circles from top) were also calculated. The plant height was measured after cutting shoot rooted on the MS culture medium for 30 days and 30 days after transplanting them into pots, respectively. The other dates were measured form 60-days olds plants planted into pots. Besides, the image-analyzing program Image J (http://rsb.info. nih.gov/ij/) was adapted to measure the length, width and area of compound leaves. Averages and standard errors were calculated from eight different plants.

### Anatomical analyses of the leaf

Sectioning of leaf was carried out by hand on mature fresh tissues of 90-day-old plants. Fine cut, with the help of a sharp razor blade and instantly fixed by 70% ethanol/acetic acid/40% formaldehyde (18:1:1, by volume; FAA), dehydrated in gradient ethanol–water series, then fixation, sectioning and dew axing. Cut samples along the middle to prepare the transverse sections of leaves. Finally, we visualized anatomical structure of leaf under a microscope (OLYMPUS IX71) and photographed. The areas of cells and average cell number per area (10000 μm^2^) were quantified by Image J software (http://rsbweb.nih.gov/ij/).

### Quantification of endogenous bioactive gibberellin contents

Collected apical shoots stem and frozen in liquid nitrogen then used for GA_3_ determination immediately. We used gibberellin (GA_3_) kit (GA-4-Y Comin Biotechnology Co., Ltd., China) and IAA (IAA-4-C Comin Biotechnology Co., Ltd., China)to extract and purify GA_3_ and IAA. The HPLC (High Performance Liquid Chromatography) was used to measure the concentration of endogenous bioactive gibberellin (GA_3_) and IAA in apical shoots stem of wild type and *MED18*-RNAi lines. Moreover, the experimental operation is strictly related to gibberellin (GA_3_) kit instruction manual.

### Statistical analysis

Data were displayed as mean ± standard deviation. Significant differences between *SlMED18*-lines and wild type were analyzed by the t-test (**P < 0.01 and *P < 0.05). Considering the biological significance of the differential expression, we used the mean value of the standard deviation (SD) with three biological repeats to represent the measured value.

## Electronic supplementary material

Supplementary Material
