# Supplementary material for: Silencing SlMED18, tomato Mediator subunit 18 gene, restricts internode elongation and leaf expansion
Source: Sci Rep. 2018 Feb 19;8:3285. doi: 10.1038/s41598-018-21679-1 (PMC5818486; doi:10.1038/s41598-018-21679-1)
Supplement: Supplementary file 1 — Supplementary Material [file 41598_2018_21679_MOESM1_ESM.doc]

**Supporting information**

**Silencing SlMED18, tomato Mediator subunit 18 gene, restricts internode elongation and leaf expans**

Yunshu Wang, Zongli Hu, Jianling Zhang, XiaoHui Yu, Jun-E Guo, Honglian Liang, Changguang Liao, Guoping Chen*

Laboratory of molecular biology of tomato, Bioengineering College, Chongqing University, Chongqing, People’s Republic of China

*Corresponding author: Guoping Chen

Bioengineering College, Chongqing University, Chongqing, People’s Republic of China

Phone: 00862365112674

Fax: 00862365112674

E-mail: chenguoping@cqu.edu.cn

**Supplementary Table S1. Primers for PCR amplification and quantification. All the primers we used were designed by Primer premier 5.0 software.**

| Primer names | Sequences |
| --- | --- |
| *SlMED18*-F | 5' TCACTTGGCTACAAGCTAGACC 3' |
| *SlMED18*-R | 5' TTCACTTTGTGCCTCCACC 3' |
| *NPTII*-F | 5' GACAATCGGCTGCTCTGA 3' |
| *NPTII*-R | 5' AACTCCAGCATGAGATCC 3' |
| *qSlCAC*-F | 5' CCTCCGTTGTGATGTAACTGG 3' |
| *qSlCAC*-R | 5' ATTGGTGGAAAGTAACATCATCG 3' |
| *qSlMED18*-F | 5' ATATCTTGCACCGCTCCTTCA 3' |
| *qSlMED18*-R | 5' GGGAAGGAGAATGGCGAAATA 3' |
| *qSlCPS*-F | 5' AGGTCTTGTTTTGGCTCCCC 3' |
| *qSlCPS* -R | 5' CAAGTAGTGATGGATGTCTCTGCC 3' |
| *qSlKAO*-F | 5' TGGACTTACACCAAAGGTAGGAA 3' |
| *qSlKAO*-R | 5' AAATACATCACTGGACAAGACGG 3 |
| *qSlGA20ox1*-F | 5' TTCTCAAATTGGCTTCATGATCAA 3' |
| *qSlGA20ox1*-R | 5' TTCCCCCTAATTCCCATAACAT 3' |
| *qSlGA3ox2*-F | 5' GTAGACCAAAGGAACCCTCAAAT 3' |
| *qSlGA3ox2*-R | 5' GCCGAACAGATGAAAGTGCT 3' |
| *qSlGID1-A*-F | 5' GCGGTGTTGTTGAATGAGAATC 3' |
| *qSlGID1-A*-R | 5' GTCTTGTGCAGATCAGCTCCC3' |
| *qSlGAST1*-F | 5' CAACAACAGAGAAATAACCAAC 3' |
| *qSlGAST1*-R | 5' TTATACGATGTCTTTGAACACC 3' |
| *qSlPIN1*-F | 5’ GCTGCAGGCTGGTCTAGATT 3’ |
| *qSlPIN1*-R | 5’ AACAATGGCAACAAAGCACA 3’ |
| *qSlPIN4*-F | 5' AAAGAGGGACCCACTGGACT 3' |
| *qSlPIN4*-R | 5' TCATAACACTAGCCGGAGGC 3' |
| *qSlLAX1*-F | 5’ GTTTACTGGGCGTTTGGAGA 3’ |
| *qSlLAX1*-R | 5’ TAACGGTGTACACGCGAATC 3’ |
| *qSlLAX4*-F | 5’ GATCCCAGTCGGTGTTCCTA 3’ |
| *qSlLAX4*-R | 5’ GATGGGCCAGTATGAGCAAT 3’ |
| *qSlIAA3*-F | 5’ GCCACCAGTTCGATCATACA 3’ |
| *qSlIAA3*-R | 5’ ATAAGGTGCTCCATCCATGC 3’ |
| *qSlIAA14*-F | 5' GTGATGATATTTCTTGTGCTATGGAG 3 |
| *qSlIAA14-*R | 5' ATGATCTCACTGGTGGCCAAC 3' |
| *qSlARF8*-F | 5' TGACATCGAATGGAAATTCAG 3' |
| *qSlARF8*-R | 5' GTCTCTTAGCACTAACAAACAC 3' |
| *qSlARF19*-F | 5'CGTTCTTTAGATCAAAGCGGC 3' |
| *qSlARF19*-R | 5' CATCCATTGGACTAAGCTCAGG 3' |
| *qSlLA*-Q-F | 5' GAGTCTGATTCAATGGCATACACC 3' |
| *qSlLA*-Q-R | 5' GCAAACAGCAGCAATATGACAAG 3' |
| *qSlKNOX1*-Q-F | 5' AGATGTTTCCAAGGACCCAGAAC 3' |
| *qSlKNOX1*-Q-R | 5' CCGAACGGGAGCATTACCA 3' |
| *qSlKNOX2*-Q-F | 5' TTGTTGCGTAAGTACAGCGGT 3' |
| *qSlKNOX2*-Q-R | 5' CAGTTGTTTATTTGCTTCTGGTCC 3' |
| *qSlPHAN*-Q-F | 5' AGATTAGGAAAGTGGTGGGAAGT 3' |
| *qSlPHAN*-Q-R | 5' AGCCATAAGTAAACCTGGGACA 3' |

**
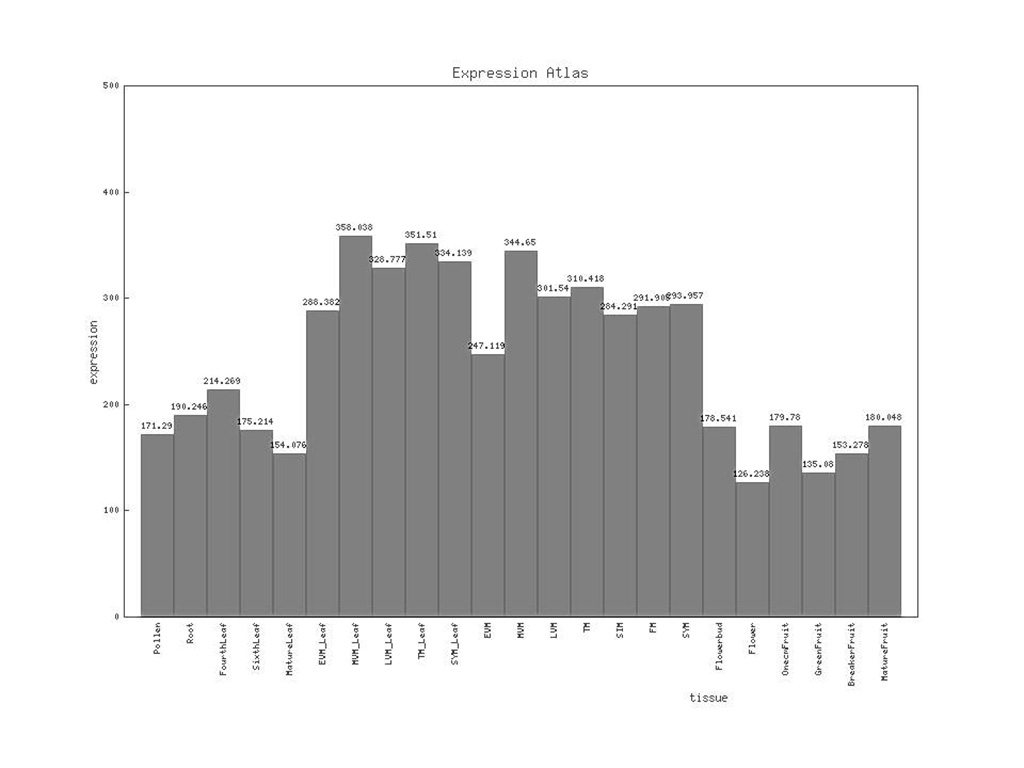
**

**Supplementary Figure S1.** Expression analysis prediction of SlMED18.  The bar graph of expression profile in various wild type tomato tissues obtained from Tomato lab website. The tissues included pollen; root; fourth Leaf; sixth Leaf; Matura Leaf; fifth leaf initiated (EVM); sixth leaf initiated (MVM); seventh leaf (LVM); eighth leaf initiated (TM); sympodial inflorescence meristems (SIM); flower meristem (FM); sympodial shoot meristem (SYM); Flower bud; Flower; One cm Fruit; Green Fruit; Breaker Fruit; Mature Fruit. The number above the histogram represents relative expression values in various wild type tomato tissues and the 0 means no expression.

**
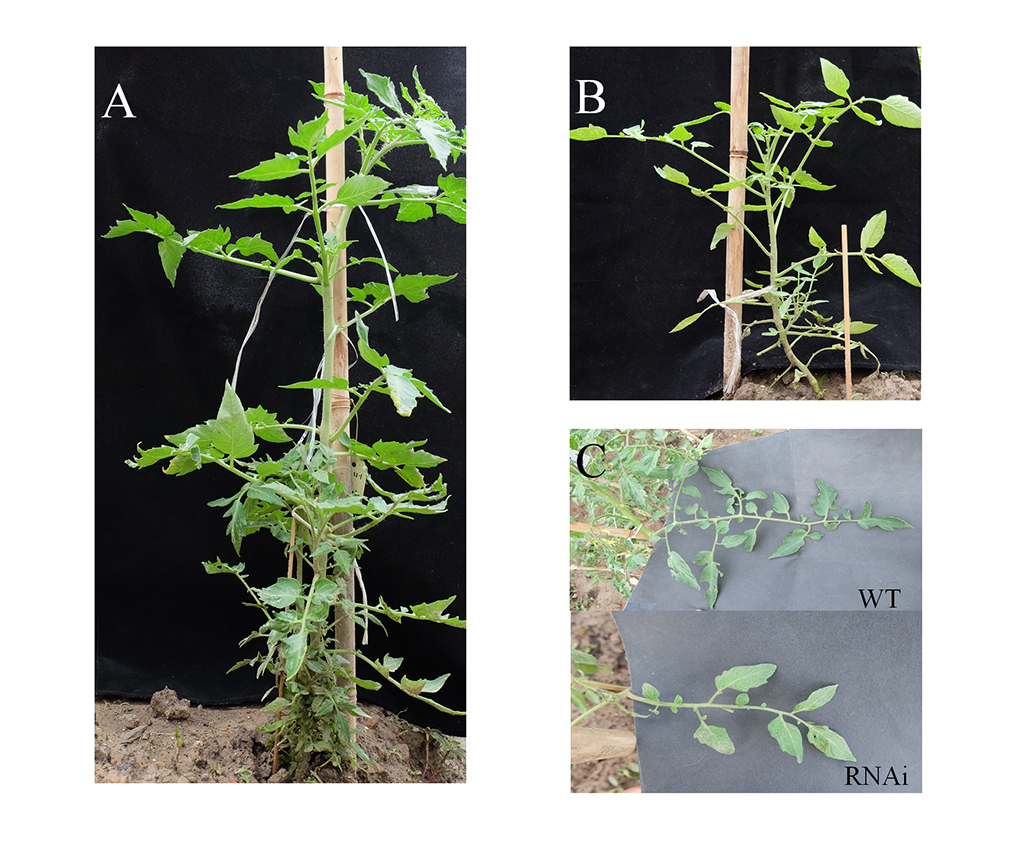
**

**Supplementary Figure. S2.** Silencing *SlMED18* restricts internode elongation and leaf expansion. The growth situation of 90-days old wild type plant (A) and *SlMED18*-RNAi plant (B). (C) The compound leaf of the same node (the fifth node) of 90-days old wild type and *SlMED18*-RNAi tomato plants.

**
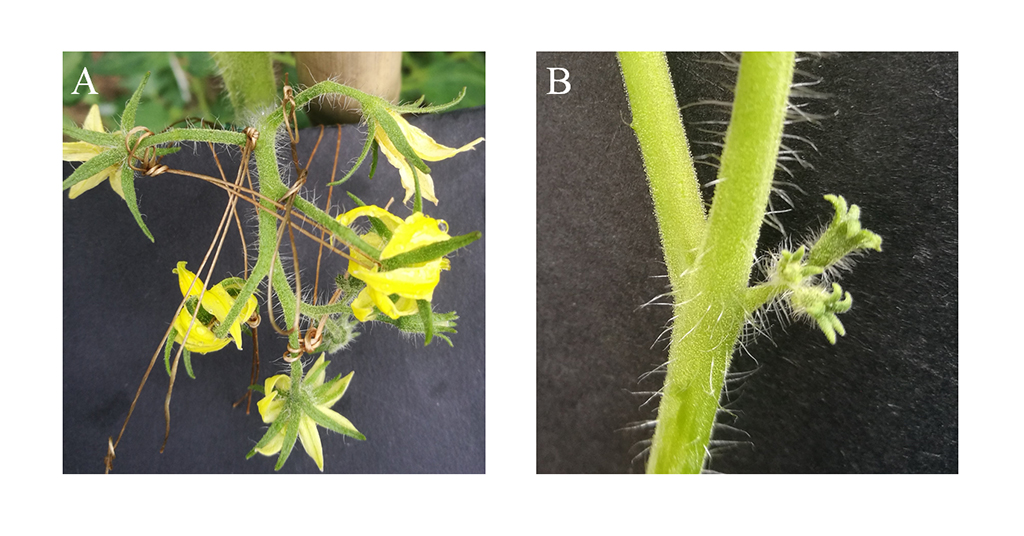
**

**Supplementary Figure. S3.** The inflorescences architecture was altered in *SlMED18* RNAi plants. The inflorescences in wild-type (A) SlMED18-RNAi plants (B), respectively. The first inflorescences were pictured.


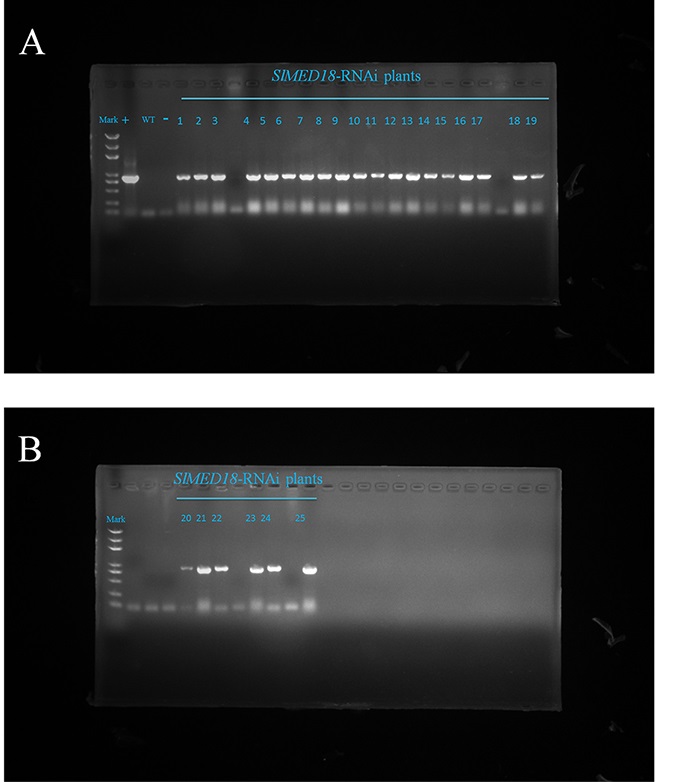


**Supplementary Figure. S4.** PCR analysis with the marker gene (NPT II) of the obtained transgenic plants. Mark: DL2000 plus marker; 1-25: the obtained *MED18*-RNAi transgenic plants. pBIN19: positive control of transgenic; WT: negative control of transgenic; －: dddH_2_O blank control.
